# Anti-Inflammatory Effects of Flavonoids in Common Neurological Disorders Associated with Aging

**DOI:** 10.3390/ijms24054297

**Published:** 2023-02-21

**Authors:** Hilda Martínez-Coria, Isabel Arrieta-Cruz, Roger Gutiérrez-Juárez, Héctor Eduardo López-Valdés

**Affiliations:** 1 Department of Physiology, Faculty of Medicine, National Autonomous University of Mexico, Mexico City 04510, Mexico; 2 Department of Basic Research, National Institute of Geriatrics, Ministry of Health, Mexico City 10200, Mexico; 3 Department of Biomedical Sciences, School of Medicine, Faculty of Higher Studies Zaragoza, National Autonomous University of Mexico, Mexico City 09230, Mexico

**Keywords:** flavonoids, ischemic stroke, neurodegenerative diseases, Alzheimer’s disease, Parkinson’s disease, neuroinflammation

## Abstract

Aging reduces homeostasis and contributes to increasing the risk of brain diseases and death. Some of the principal characteristics are chronic and low-grade inflammation, a general increase in the secretion of proinflammatory cytokines, and inflammatory markers. Aging-related diseases include focal ischemic stroke and neurodegenerative diseases such as Alzheimer’s disease (AD) and Parkinson’s disease (PD). Flavonoids are the most common class of polyphenols and are abundantly found in plant-based foods and beverages. A small group of individual flavonoid molecules (e.g., quercetin, epigallocatechin-3-gallate, and myricetin) has been used to explore the anti-inflammatory effect in vitro studies and in animal models of focal ischemic stroke and AD and PD, and the results show that these molecules reduce the activated neuroglia and several proinflammatory cytokines, and also, inactivate inflammation and inflammasome-related transcription factors. However, the evidence from human studies has been limited. In this review article, we highlight the evidence that individual natural molecules can modulate neuroinflammation in diverse studies from in vitro to animal models to clinical studies of focal ischemic stroke and AD and PD, and we discuss future areas of research that can help researchers to develop new therapeutic agents.

## 1. Introduction

The most common aging-related diseases include focal ischemic stroke and neurodegenerative diseases such as Alzheimer’s disease (AD) and Parkinson’s disease (PD). Aging is a progressive, irreversible, and inevitable process that involves a distinctive decline in physiological functions and physical appearance, resulting from tissue degeneration and the dysfunction of vital organs [1]. A focal ischemic stroke is caused by the occlusion of a cerebral artery and is the most frequent type of cerebrovascular disease, and aging is its most nonmodifiable risk factor. Neurodegenerative diseases have no cure and are caused by progressive degeneration and/or neuron death to produce debilitating conditions. Neurodegenerative diseases also include Lewy body dementia, vascular dementia, amyotrophic lateral sclerosis, and frontotemporal dementia [2,3]. Meanwhile, the components of the human diet, such as vegetables, cereals, tea, wine, and fruits, contain different compounds including flavonoids, which are a class of polyphenols [4]. Many recent studies have shown that these compounds have a beneficial effect on the primary cell culture of glial and neurons and pre-clinical animal models of human focal ischemic stroke and neurodegenerative diseases. The active compounds and their mechanism of action for most flavonoids in the human diet and herbal medicine are still not well defined because they contain multiple bioactive molecules that can modulate multiple pharmacologic targets. To obtain more significant information, the scientific research has concentrated mainly on known biological activities of purified single compounds to offer an evidence base for the rationale of traditional practice but also to support their integration into modern medical practice [5].

In the sections below, we will describe the basic knowledge of natural flavonoids, neuroinflammation, aging, focal ischemic stroke, AD, and PD, and we will summarize the evidence relating to the anti-inflammatory effects of single flavonoids in different model systems of those diseases and clinical studies, and finally, we will suggest future areas of research to improve our understanding of single flavonoids molecules to help to establish more solid bases to facilitate their future use as a therapeutic alternative.

## 2. Flavonoids

**Figure 1:**
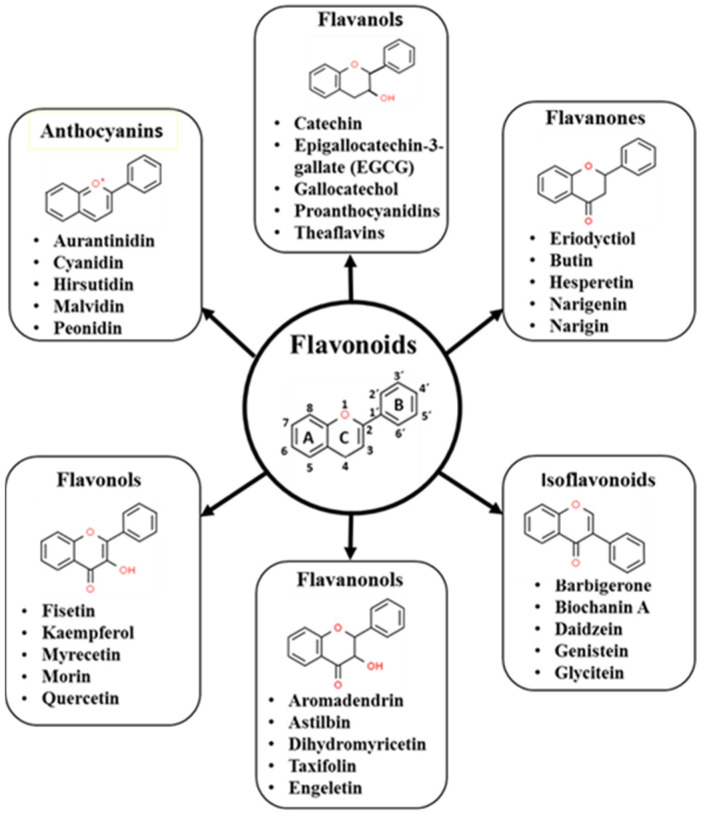
Basic flavonoid share a C6–C3–C6 structure containing two aromatic rings (A and B rings) linked by a three-carbon bridge. Some representative compounds are shown below each flavonoid subgroup.

Flavonoids are present in all vascular plants and are the most common class of polyphenols. In the plant, flavonoids are secondary metabolites that have a wide range of biochemical, physiological, and ecological functions, such as the coloration of petals and flowers, protection against ultraviolet light, and cell growth. Moreover, a single plant often contains many different flavonoids [6]. In nature, flavonoids are broadly distributed in the plant kingdom and are the main phytochemicals found in more than 6000 species of plants, and they also are abundantly found in plant-based foods and beverages, including vegetables, tea, fruits, grains roots, cocoa, and wine [7]. Flavonoids are low-molecular-weight compounds that can be divided into subclasses such as: anthocyanins, chalcones, flavanols (or catechins), flavones, flavanones, flavonols, flavanonols, and isoflavonoids (Figure 1) [7]. Except for catechins, all flavonoids contained in foods and beverages are in the form of glycosides, which must be removed to be absorbed from the small intestine upon ingestion. Flavonoid aglycones undergo conjugation reactions before passing into the bloodstream, and these reactions form sulphates, glucuronides, and/or methylates metabolites (conjugates), which can be subjected to additional phase II metabolism in the liver, and then returned to the circulatory system. Moreover, some conjugates may be exported into the bile duct or excreted by the kidney. Flavonoids not absorbed from the small intestine will be degraded in the colon [8]. Except from catechins, the rest of the flavonoids present in plasma and urine are primarily conjugated forms. Consequently, cells in the body are usually exposed to apparently less active flavonoid metabolites and conjugates, rather than aglycones [9]. However, there is evidence that deconjugation may occur in situ. An in vivo study has shown that glucuronidated quercetin metabolites were deconjugated in their aglycone form in the mesenteric vasculature of rats by the action of β-glucuronidase, and this effect was inhibited when an inhibitor of that enzyme was present [10], which suggests that deconjugation may occur in situ to produce a more effective aglycone form. The absorption and distribution of flavonoids around the body, as well as their excretion in urine, are carried out by members of ATP-binding cassette (ABC) transport systems, which translocate solutes across cell membranes [11]. The physicochemical properties (such as molecular size and lipophilicity, solubility, configuration, and pKa value) of each flavonoid determine the degree of absorption [12]. Flavonoids show great variability in the velocity and magnitude of absorption, plasma half-life, bioavailability, and plasma kinetics, but in general, they show rapid urinary and biliary excretion and low bioavailability, and it has been suggested that after the consumption of 10–100 mg of a single phenolic compound, their plasma concentration rarely exceeds 1 μM [13]. Moreover, Manach et al. [14] analyzed 97 bioavailability studies in humans and found that plasma concentrations of total metabolites ranged from 0 to 4 µmol/L with an intake of 50 mg aglycone equivalents, and the relative urinary excretion ranged from 0.3% to 43% of the ingested dose depending on the specific polyphenol. They also found that among all of the compounds analyzed, isoflavones and gallic acid had the best rate of absorption, followed by catechins, flavanones, and quercetin glycosides, and that proanthocyanidins and anthocyanins had very low bioavailability. Some evidence suggests that some flavonoids and/or their metabolites such as anthocyanins and (−)-epicatechin and some of their metabolites can cross the blood–brain barrier [15,16,17]. Flavonoids have pleiotropic effects that can produce health benefits, as shown in several diseases including cardiovascular diseases, neurological disorders, and cancer, and these benefits are seen in antiviral, antioxidant, and anti-inflammatory mechanisms, among others [6,8].

## 3. Neuroinflammation

An inflammatory reaction in the central nervous system (CNS) or neuroinflammation are generally induced when innate immune cells detect tissue damage or an infection. This response is critical to isolate damaged tissue from uninjured areas and to repair and clean the extracellular matrix. Acute inflammation is beneficial and promotes regeneration, but chronic and excessive, as well as stable, low-grade, inflammation can produce the onset or exacerbation of cell injury [3]. It is generally accepted that low-grade neuroinflammation is the main factor in the onset and development of several neurological diseases. In aging, the major source for low-grade, chronic inflammation is the accumulation of endogenous host-derived cell debris due to both increased production and impaired elimination, and also immunosenescence, but these alterations are not commonly the initiating factor of neurodegenerative diseases. However, they contribute to amplifying the disease state, which would suggest that neuroinflammation plays an important role in neuronal dysfunction and death [18].

Glial cells in the CNS carry out different activities during neuroinflammation and can promote protection or damage, depending on the particular environments of inflammation and time. All glial cells play a role in the immune response, but the most important ones are the microglia and astrocytes. Microglia is the main immune cell in the CNS. These cells continuously scan the microenvironment of the parenchyma and are the first cells to respond to the occurrence of any damage [18]. In addition to being the most numerous cell type in the CNS, astrocytes have varied homeostatic functions, for example, they control the extracellular pH, antioxidant functions, neurotransmitter uptake, and the recycling of glutamate and GABA, regulate the cerebral blood flow and the blood–brain barrier (BBB), promote synaptogenesis, supply energy metabolites to the neurons, and they form part of the innate immune system of the CNS [19].

In neurological disease, glial cells can recognize endogenous molecules released by damaged or dead cells (damage-associated molecular patterns: DAMPs) and molecules present in pathogens (pathogen-associated molecular patterns: PAMPs) through pattern recognition receptors (PRR), which are formed by several subfamilies including the Toll-like receptors (TLRs) [18,20]. TLRs are type 1 transmembrane glycoproteins that are extensively expressed in microglia and astrocytes, with specific subtypes expressed in neurons and oligodendrocytes [21]. Each TLR subtype recognizes different PAMPs or DAMPs, for example, TLR4 recognizes lipopolysaccharide (LPS), and also, accumulated, misfolded proteins, including Aβ and α-synuclein present in AD and PD, respectively [18]. DAMPs comprise an extensive range of molecules such as uric acid, cytokine IL-1α, ATP, and nuclear and cytoplasmic proteins released during necrosis, and it has also been suggested that some members of the extended IL-1 cytokine family including IL-1β, IL-18, IL-33, IL-36α, IL-36β, and IL-36γ also act as DAMPs and stimulate the sterile inflammation induced by necrosis [22]. The interaction of ligands with the TLR of the host cell activates an intracellular signaling cascade that causes the release of inflammatory cytokines and other immune modulators as a protective mechanism and to repair the damaged tissue. However, excessive TLR activation disrupts immune homeostasis, causing constant proinflammatory cytokine and chemokine production, which contributes to the development and progression of many diseases [21]. After the initial interaction of the ligand and TLR, the latter one activates one of the several signal transduction pathways such as phosphoinositide 3-kinase/protein kinase B (PI3K/AKT), mammalian target of rapamycin (mTOR), or mitogen-activated protein kinase (MAPK), which leads to the activation of different transcription factors such as activator protein 1 (AP-1), nuclear factor kappa-B (NF-κB), nitric oxide synthase (iNOS), interferon regulatory factor 3 (IRF3), and cyclooxygenase-2 (COX-2), which mediate the production of proinflammatory cytokines, chemokines, and inducible enzymes, all of which result in neuroinflammation [23,24]. The activation of the PI3K/AKT/mTOR pathway can activate the transcription factor NF-κB and induce the expression of proinflammatory molecules (e.g., IL-6 and TNF-α), iNOS, and COX-2, while other TLRs can activate the MAPK (p38 MAPK or SAPK/JNK) pathway, which then activates the transcription factor AP-1 and promotes the expression of proinflammatory molecules, including cytokines (e.g., IL-6, iNOS, and COX-2) [24]. The NOD subfamily is another well-known PRR member, which is part of the inflammasome NLRP3, together with the adaptor protein apoptosis-associated speck-like protein comprising a caspase recruitment domain (ASC) and procaspase 1, which together form an oligomer when the stimuli activate the receptor, and thus, induce the active precursors of proinflammatory cytokines, such as IL-1β and IL-18 [25]. Cytokines are the main communication mechanism used by the immune system and consist of polypeptides and glycoproteins synthetized by immune cells such as chemokines, lymphokines, interleukins (IL), tumor necrosis factor (TNF), and interferons (IFN), which can act as pro- and anti-inflammatory molecules [26]. Cytokines can have both anti- and proinflammatory effects. Anti-inflammatory cytokines include: IL-4, IL-6, IL-10, IL-11, IL-13, IL-1 receptor antagonist (IL-1RA), and TGF-β, while the proinflammatory cytokines include IL-1β, IL-6, IL-8, IL-12, TNF-α, and interferons, among others [26]. After secretion to the extracellular space, these molecules interact with cytokine receptors to initiate cytokine intracellular signaling, which modulates a diverse range of biological functions, and most cytokine receptors activate the JAK-STAT pathway [27]. The primary proinflammatory cytokines, such as TNF-α, IL-1α, and IL-6, contribute significantly to inflammaging in healthy elderly people and play a major role in several age-related diseases, including neurodegenerative diseases [22]. Furthermore, TNF-α also induces apoptosis by activating receptors such as tumor necrosis factor receptor 1 (TNFR1), 2 (p75), and CD95 (APO-1/Fas), which contain a homologous cytoplasmic sequence identifying an intracellular death domain [24]. Microglia and astrocytes can release anti- and proinflammatory cytokines, but the type of secretion seems to be related to the specific phenotypes of the glial cells, their interaction with each other, and the specific context. Glial cells show different phenotypes that are the result of transcriptional and functional changes that are generally known as activation or reaction ones. Several different phenotypes have been observed, but M1 for harmful and neurotoxic inflammation functions and M2 for pro-reparative and anti-inflammation functions are considered to be opposite states of reactive microglia, but the microglia can switch between these two phenotypes according to different environments [28]. Similarly, for astrocytes, the A1 phenotype is toxic to neurons and oligodendrocytes, while the A2 phenotype is protective [29,30]. In human neurodegenerative illnesses such as PD and AD, M1 microglia, as well as astrocyte A1, are highly present, and it has been proposed that M1 microglia induces the A1 astrocyte phenotype in these illnesses [31]. M1 microglia and A1 astrocytes secrete proinflammatory cytokines, which can induce apoptosis through the activation of extrinsic pathways and also induce the overproduction of ROS, mitochondrial dysfunction, DNA damage, and the production of more inflammatory mediators that contribute to cell aging, and also, induce the permeabilization of the BBB [32,33]. Most types of CNS diseases have a neuroinflammatory component, and activated microglia and astrocytes have been found [18].

## 4. Aging

Aging is a complex and inevitable process that results from the interaction of environmental epigenetic and genetic factors that produce a gradual reduction of homeostasis with age. This change is characterized by the progressive degeneration of tissue and functions in several organs, which contributes to increasing the risk of disease and death [34,35]. In the brain, aging affects different cell types and regions differently, and the individual variability is broad, but in general terms, there is a reduction of white and grey matter density, volume loss, cortical thinning, and the atrophy of specific brain regions, including the hippocampus [36]. Age-related diseases or aging-related diseases group different illnesses together, including arthritis, cancer, hypertension, type 2 diabetes, focal ischemic stroke, and many neurodegenerative diseases such as AD and PD. In normal aging, alterations occur in the nervous system to produce functional and structural changes. Functional changes include a decrease in blood flow and a reduction of synapses and neurotransmitter release and several areas, but the area that is most commonly affected is the hippocampus. On the other hand, structural changes include the enlargement of the ventricles, cerebral atrophy, and neuronal loss [37]. It is important to note that endothelial dysfunction is a significant contributor to cerebrovascular aging, which promotes oxidative stress and neuroinflammation in age-related disorders such as dementias and focal ischemic stroke [38]. There is a noticeable change in altered intercellular communication known as ‘inflammaging’, which is characterized by a process of chronic and low-grade inflammation, and a general increase in the secretion of proinflammatory cytokines and inflammatory markers [39]. Additionally, another feature is the presence of cellular senescence in tissues and organs, including the immune system (immunosenescence), producing reduced humoral and cellular responses for both the innate and adaptative immune systems. Senescent cells show increased activity of senescence-associated β-galactosidase (SA-β-GAL), a failure to re-enter the cell cycle in response to mitogenic stimuli, resistance to cell death, and a proinflammatory secretome called senescence-associated secretory phenotype (SASP) [32]. Some alterations such as neuroinflammation, DNA damage, and oxidative stress that are present in neurodegenerative diseases and focal ischemic stroke can induce cellular senescence, and it has been suggested that cellular senescence contributes to the pathophysiology of these disorders [40,41].

Many of the inflammaging characteristics can be found in the blood, for example high levels of proinflammatory cytokines (e.g., IL-1β, IL-6, IL-8, and IL-10) in sera or plasma [26], monocytes and macrophages with impaired phagocytosis and ability to heal injuries, the presence of senescent T cells, impaired macrophage polarization, and antibody production by the activated B cells [42]. In age-related diseases, systemic inflammation may contribute to neuroinflammation because circulating proinflammatory molecules can interact with endothelial cells from cerebral vasculature and induce the release of more cytokines to cause BBB impairment [43]. Oxidative stress is another factor present in neurodegenerative age-related diseases. This alteration is due to the excessive production of unstable molecules that contain oxygen called reactive oxygen species (ROS), and also, because the antioxidant system in the cells is not able to neutralize them. This mitochondrial dysfunction produces DAMPs in the cell, which initiate several inflammatory cascades, causing the activation of the innate immune system and NLRP3 inflammasome, which results in chronic inflammation [18].

## 5. Basic Physiopathology of Focal Ischemic Stroke, AD, and PD

### 5.1. Focal Ischemic Stroke

Ischemic strokes or cerebrovascular accidents are the most frequent types of cerebrovascular diseases and are one of the main causes of death and disability worldwide [44]. The main type of strokes is the focal ischemic, which is due to an obstruction of the arterial blood flow to a specific brain region [45]. Aging is considered to be the most important nonmodifiable risk factor, and the risk of a stroke occurring doubles every 10 years after the person reaches the age of 55, and approximately three quarters of all strokes occur in persons aged ≥65 years [46,47]. It is important to highlight that modifiable risk factors such as diabetes and high blood pressure steadily increase with age, and it has been shown that these chronic conditions are very highly prevalent in persons that have suffered strokes [47]. In most countries, the approved treatments for an acute focal ischemic stroke are the endovascular thrombectomy and intravenous recombinant tissue plasminogen activator (rtPA), which focus on removing the occlusion in the artery and saving the penumbra cells to reduce the infarct core enlargement [45], but several conditions must be met to obtain the therapeutic benefits. Endovascular thrombectomy shows benefits when it is applied within the first six hours and rtPA shows benefits when it is applied within the first four and a half hours of stroke onset [48].

The cessation of or decrease in cerebral blood flow may produce different levels of damage depending on several factors such as the time elapsed, cell resistance, and the magnitude of the ischemia, which results in the activation of very complex cascades of cellular and molecular events with a temporal overlapping profile that evolves over minutes, hours, or days, inducing transient-to-irreversible injuries (e.g., cell death) in all cell types and damage. The pathologic cascades produce damage in two different areas: the ischemic core and the penumbra. In the ischemic core, the abrupt decrease in cerebral blood flow leads to permanent damage in the cell and rapid cell death by necrosis. The size of the necrotic area will depend mainly on the location of the stroke, its duration, and its magnitude. The penumbra surrounding the core area is perfused by collateral blood vessels, which help to keep the cell structures intact, but functionally weakened [45]. After a few minutes of cerebral blood vessel occlusion, the first pathological event is activated due to the reduction of oxygen and glucose, which leads to a failure to produce high-energy molecules to maintain cellular homeostasis. This event sets off several mechanisms, which include ionic imbalance, cytotoxic and vasogenic edemas, excitotoxicity, calcium overload, excitotoxicity, oxidative and nitrosative stress, peri-infarct depolarization, blood–brain barrier (BBB) disruption, apoptosis, and inflammation [45,49]. Inflammation responses occur after the damaged cells release DAMPs, which interact with Toll-like receptors in the microglia and astrocytes. After this interaction, the microglia become reactive and accumulate at the lesion core and penumbra. During the first hours after the damage, these reactive microglia have an anti-inflammatory profile, but after this period, they switch to a proinflammatory profile, and several of the proinflammatory mediators (e.g., reactive oxygen species, cytokines, and tumor necrosis factor-α) released can induce astrogliosis from 4 to 24 h after stimulation [50,51,52]. M2 microglia found around the lesion site migrate towards the lesion core and in the penumbra, and following cell death, they begin the phagocytic removal of cell debris [53,54]. Proinflammatory cytokines (e.g., IL-1, IL-6, and TNF-α) released by M1 microglia promote a gradual alteration in the BBB and allow the infiltration of circulating leucocytes, which eventually release proinflammatory cytokines (e.g., interleukin-1β and interferon-γ) to damage the cell structures directly or indirectly and contribute to the enlargement of the lesion [55,56,57]. Cytokines released by M1 microglia and other glial cells and neurons promote astrogliosis, and these reactive astrocytes participate in many protective mechanisms after a stroke (e.g., neurotransmitter uptake, pH regulation, the anti-inflammatory release of cytokines, and glial scar formation), but they also have detrimental effects including the release of several detrimental factors such as proinflammatory cytokines (e.g., TNF-α and IL-1), matrix metalloproteinases (e.g., the degradation of the matrix protein), and proteoglycans (e.g., these cause inhibition of axon regeneration and myelination), which can contribute to expanding the lesion and/or decreased recovery [58,59]. Around 6 days after the injury, glial scar formation starts, and involves a subset of reactive astrocytes and other cells (e.g., reactive microglia and NG2 cells) and is completed between 2 and 4 weeks after the stroke [60,61]. Even after the glial scar, which serves as a protective barrier, has been formed, the cells in the penumbra are still exposed to several deleterious mechanisms, such as vasogenic edema, apoptosis, astrogliosis, and inflammation, which can last many months or even years. However, neurons and other cell types are viable and have a long-term potential for remodeling the tissue and forming new circuits to sustain a functional recovery [45,62], and this also the main target for any therapeutic interventions.

### 5.2. Alzheimer’s Disease (AD)

AD is a progressive disease that is generally manifested by a loss of memory and difficulties with communicating, behaving correctly, and using problem-solving skills. AD is a neurodegenerative disorder that results in neuronal loss and brain atrophy in extensive areas of the hippocampus and cerebral cortex, synapse loss, and ultimately, death [63]. AD is generally divided into familial AD (FAD) and idiopathic or sporadic AD (SAD). FAD is caused by dominant genetic mutations in presenilin 1 (*PSEN1*), presenilin 2 (*PSEN2*), and amyloid-beta A4 precursor protein (*APP*), and it accounts for 3% of the reported cases of AD. SAD has no single genetic cause, and it represents over 95% of all cases. Since age is considered to be the main risk factor for AD, there is another classification that takes into account the age at which the disease began, and this is divided into early-onset and late-onset types. Early onset occurs before the age of 65, and most cases are FAD ones. Most late-onset cases are SAD one, and it has a mean onset age of 80 years [64,65]. In addition to *APP*, *PSEN1*, and *PSEN2* genes, the ε4 allele of apolipoprotein E (*APOE*) is another genetic risk factor for AD. APOE is a protein related to lipid metabolism and is immunochemically colocalized to vascular amyloid deposits, neurofibrillary tangles (NFT), and senile plaques in AD [66]. AD is related to the accumulation of insoluble forms of amyloid-β (Aβ) in plaques and the intraneuronal deposition of neurofibrillary tangles (NFT), which are composed of hyperphosphorylated tau protein. The amyloid hypothesis of AD suggests that alterations to APP metabolism and Aβ accumulation are the main events in AD [67]. On the other hand, the tau hypothesis of AD suggests that aggregates of misfolded and fibrillar hyperphosphorylated NFT accumulate inside the neurons and propagate through cells in a prion-like way, eventually disseminating into the brains of AD patients. The neuropathologic hallmarks of AD are Aβ plaques and NFTs, but generally, they are accompanied by neuronal and synaptic loss, reactive astrocytes, microglial activation, the blood–brain barrier alterations, and brain atrophy [68]. Moreover, it is noteworthy that a high percentage of patients with AD also have cerebral amyloid angiopathy, a condition characterized by an accumulation of amyloids in the cerebral vasculature, which can lead to intracerebral hemorrhages and microbleeds, and these events accelerate AD [64]. In AD, the microglia and astrocytes play an important role in the neuroinflammatory response, and also, in the development of the disease. Aβ can interact with the microglia through the NLRP3 inflammatory complex and CD36-TLR4-TLR6 receptor complex, causing immune responses, cell damage, and the release of inflammation-inducing factors, such as IL-1β and TNF-α. Moreover, high levels of proinflammatory cytokines IL-1β and Il-6 are elevated in the peripheral blood of AD patients [69]. Activated microglia release the proinflammatory cytokines IL-1α, C1q, and TNF-α that can induce the A1 proinflammatory astrocytes, which can produce a secondary inflammatory response [31]. The evidence from postmortem analyses of AD patients shows alterations in microglia morphology such as reduced branching and arborized areas and immunoreactivity to ionized calcium-binding adaptor molecule 1 (Iba 1), which is a microglia/macrophage-specific protein that is upregulated in activated microglia [70]. Activated microglia were observed in the entorhinal, temporoparietal, and cingulate cortices in positron emission tomography (PET) studies in humans. These PET studies use the selective marker 11PK11195, which labels the target translocator protein (TSPO) on the external mitochondrial membrane that increases its density in active microglia and the regions [71]. Additionally, the occurrence of microglia activation was confirmed using different markers alone (e.g., 11PK11195 and 11 PBR28) or in combination with tau (e.g., 18 F-AV145) or Aβ plaques markers (e.g., 18 F-flutemetamol). One study found that AD patients have increased microglial activation and amyloid in the frontal, temporal, parietal, occipital, and cingulate cortices [72], while another study found activated microglia in the occipital lobe in AD patients [73]. Additionally, it was reported that microglial activation, tau aggregation, and amyloid deposition were found in similar areas of the association cortex [74]. A longitudinal PET study (14 months) evaluated the change in microglia activation in AD patients with mild cognitive impairment (MCI), and the results show that patients with MCI demonstrated reduced activated microglia levels, which is in contrast to AD patients with more activated microglia [75]. Another study used PET (11PK11195) in combination with structural magnetic resonance imaging (structural MRI) to investigate brain atrophy, and they found activated microglia in the anterior temporal region, tau in the temporoparietal area, and grey matter atrophy [76]. A similar study found that activated microglia in temporal cortices are related to parieto-occipital atrophy and cortical thinning [77]. All of these studies confirm the presence of activated microglia in patients with AD.

### 5.3. Parkinson’s Disease (PD)

PD is an age-related neurodegenerative disease with a multifactorial etiology that causes tremors at rest, bradykinesia symptoms, and rigidity, and it shares these characteristics with other clinical syndromes referred to as “Parkinsonism” disorders [78]. The other main characteristics of PD are the degeneration of neurons in the substantia nigra pars compacta (one of the basal ganglia), intraneuronal protein aggregates called Lewy bodies, and Lewy neurites [79]. In addition to those mentioned, PD patients may also present other alterations such as sleep disorders, depression, and dementia [80]. A few cases of PD seem to have a genetic origin, and the majority of them correspond to the idiopathic form, for which the risk factors include aging and behavioral and environmental factors such as a history of melanoma, traumatic brain injury, and exposure to pesticides [79]. The monogenic mutation in several genes that encode different proteins constitute the genetic forms of PD, including the α-synuclein, in which gene duplications and triplications, as well as mutations, have been found, and this comprises the main component of Lewy bodies [79]. In physiological conditions, the neuronal protein α-synuclein participates in several activities, such as dopamine synthesis and vesicle trafficking, and it has two conformations, a soluble unfolded monomer and a multimeric membrane-bound helical α-synuclein. Meanwhile, in pathological conditions, the soluble unfolded monomer forms β-sheet-like oligomers named protofibrils, which transform into amyloid fibrils and ultimately deposit into Lewy bodies. Moreover, the protofibrils and fibrils may propagate by a transcellular mechanism from neuron to neuron [79]. Lewy body inclusions, in addition to being present in neurons of the substantia nigra, also are present in structures such as raphe nuclei, the basal nucleus of Meynert, the neocortex, and the amygdala, and also, in oligodendrocytes from the midbrain and the basal ganglia [81].

The contribution of neuroinflammation to cellular damage in PD has been confirmed in several studies. A positron emission tomography (PET) study in PD patients using a marker for active microglia showed that the rate of the activation of the microglia was increased in the substantia nigra, the caudate nucleus, the pre- and postcentral gyrus, the frontal lobe, and the putamen, which agrees well with the known distribution of neuropathological changes [82]. Additionally, increased proinflammatory mediators (e.g., TNF-β) and active astrocytes and microglia have been found in the substantia nigra in post-mortem studies on PD patient brains [83,84,85]. Moreover, the animal model of PD MPTP (1-methyl-4-phenyl-1,2,3,6 tetrahydropyridine), as well as human brains, exhibited an increase in the proinflammatory molecules (e.g., COX-2) in dopaminergic neurons from the substantia nigra [86]. Furthermore, some evidence suggests that the adaptive immune system also participates in the pathology, for example, the presence of CD4^+^ and CD8^+^ T cells in the substantia nigra from postmortem studies of PD patients and animal models [87]. Neuronal cell culture studies also show that IL-1 increases the α-synuclein [88] and that α-synuclein induces microglial activation and increases the production of TNF and IL1β [89]. When it is analyzed together, this information shows that inflammatory responses are involved in the pathophysiology of PD.

**Figure 2:**
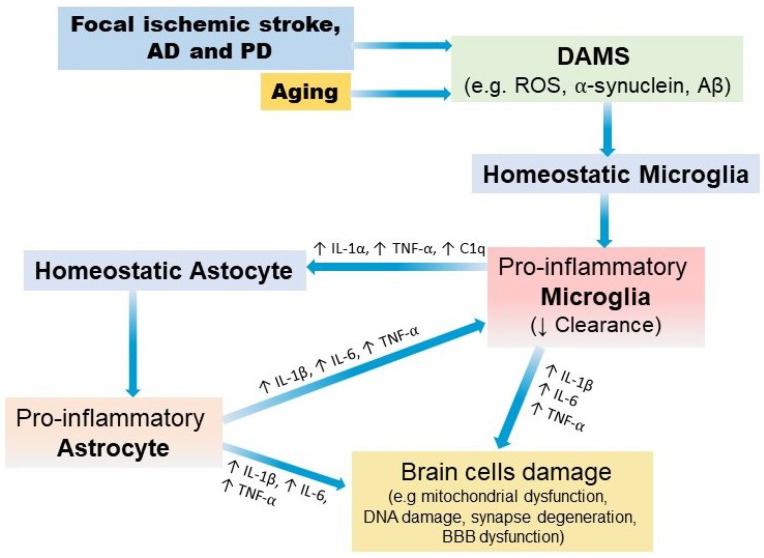
Basic neuroinflammatory responses. In homeostatic conditions, microglia and astrocytes support several brain cell functions, but during aging and in brain diseases, they change their morphology and secretome. Damage-associated molecular patterns (DAMPs) released in pathologic conditions interact with homeostatic microglia, and specific signaling occurs to induce a pro-inflammatory phenotype, which results in a decreases phagocytic effect and the release of several molecules that change homeostatic astrocytes functions towards a proinflammatory state to exacerbate inflammation and worsened the functional recovery.

Figure 2 show the main characteristic of inflammatory responses that share focal ischemic stroke, AD and PD.

## 6. Anti-Inflammatory Effects of Flavonoids in Focal Ischemic Stroke, AD, and PD

The general neuroprotective and neuroplastic effects of flavonoids are related to anti-inflammatory activity, which can increase the protection of neurons and glial cells against neurotoxins-induced injury and improve the endogenous mechanism of neuroplasticity to gain CNS functions. The effects of flavonoids in the modulation of molecular pathways involved in neuroinflammation have been mainly described in cell cultures using primary cells exposed to oxygen-glucose deprivation as the main model of ischemic stroke and lipopolysaccharides as a model of neuroinflammation in neurodegenerative disease, but animal models and clinical studies have also provided strong evidence of the beneficial effects of flavonoids. In the next sections, you will find a narrative description of the anti-inflammatory effects of the most used flavonoids on each disease in cellular, animal models, and clinical studies.

### 6.1. Anti-Inflammatory Effects of Flavonoids in Focal Ischemic Stroke

#### 6.1.1. In Vitro Studies

Oxygen glucose deprivation (OGD) is an in vitro model used for the study of the cellular and molecular pathway associated with stroke, and usually, the cells are incubated in a glucose-free medium in a deoxygenated atmosphere for different durations of exposure to these conditions depending on the preparation. Sometimes, after the OGD, the cells are returned to the pre-deprivation conditions to model the focal ischemic reperfusion injury that occurs after the blood supply is restored, and this variant is called OGD/R [90,91]. Myricetin showed a significant protective effect against inflammation in human brain microvessel endothelial cells (HBMECs) in OGD/R conditions by decreasing the number of proinflammatory cytokines such as TNF-α, IL-1β, and IL-6 [92]. Quercetin inhibits inflammation that is mediated by TLR4/MyD88/NF-κB, signaling BV2 microglial cells in mice [93]. Isoquercetin, a glucoside derivative of quercetin, has neuroprotective effects in rat cortical neurons in OGD/R conditions by inhibiting the protein expression of TLR4 and nuclear NF-κB and the mRNA expression of TNF-α and IL-6 [94]. Moreover, in rat hippocampal neurons in OGD/R conditions, isoquercetin inhibits the activation of Toll-like receptor 4 (TLR4), nuclear factor-kappa B (NF-κB), and caspase-1; the phosphorylation of ERK1/2, JNK1/2, and p38 mitogen-activated protein kinase (MAPK); the secretion of tumor necrosis factor-α (TNF-α), interleukin-1β (IL-1β), and IL-6 [95]. Furthermore, cortical neurons in similar conditions inhibit the protein expression of TLR4 and NF-κB and the mRNA expression of TNF-α and IL-6 [94]. Baicalin could effectively downregulate the expression of the NOD2 receptor (protein associated with inflammatory reactions) and TNFα at both the mRNA and protein levels in BV2 microglial cells in OGD conditions [96], inhibit the NLRP3 inflammasome IL-1β, and IL-18 expression in cortical neurons in OGD/R conditions [97], and also, decrease the secretion of TNF-α, IL-1β, IL-6 and inhibit the NF-κB signaling pathway in the brain microvascular endothelial cells (BMECs) in OGD conditions [98] and the BV2 microglia cell line in OGD/R conditions [99]. Moreover, baicalin inhibits the proinflammatory microglial polarization through the inhibition of the TLR4/NF-κB pathway and the downregulation of phosphorylated STAT1 in a microglia-neuron co-culture system in OGD conditions [100] and in human brain microvascular endothelial cells (HBMEC) in OGD conditions, it inhibits the expression of TLR4, MYD88, and p-NF-κB and decreases the release of inflammatory factors IL-6, IL-1α, IL-1β, IL8, and TNF-α [101,102]. Baicalin also decreases the release of TNF-α, IL-1β, IL-6, and IL-8, and Tlr4 mRNA expression in microglia in OGD conditions [102]. Icariin applied before the OGD/R-reduced protein level expression of IL-1β, IL-6, and TNF-α in OGD/R conditions injured the microglia [103]. Casticin reduced the expression of TLR4, NF-κB p65, and NF-κB p50 in PC12 cells in OGD/R conditions [104]. Pratensein in HT22 cells in OGD/R conditions suppresses NLRP3 inflammasome activation through Nrf2 activation, resulting in reduced inflammatory responses [105]. Tectorigenin inhibited ROS inflammatory cytokines IL-1β, IL-6, and TNF-α production in OGD/R-induced HT-22 cells [106]. Astilbin inhibits NLRP3 inflammasome and decreases the release of IL-1β and IL-18 in PC12 cells in OGD conditions [107]. Anthocyanin significantly reduced the secretion of TNF-α, IL-1β, and IL-6 in SH-SY5Y cells exposed to OGD [108]. In the mouse neuroblastoma cells N2a, tricin, an O-methylated flavone, decreases the expression of TNF-α, IL-6, and IL-1β [109]. Diosmetin inhibits the NLRP3 inflammasome pathway and inflammatory cytokines IL-1β and IL-18 in PC12 cells in OGD/R conditions [110]. Schaftoside inhibits the expression of TLR4, IL-1β, IL-6, and TNF-α in OGD-simulated BV2 microglia [111].

#### 6.1.2. Animal Model

The most commonly used animals in preclinic stroke research are rats and mice. Animal models for ischemic stroke can be divided into global, focal, and multifocal ones. There are several global ischemic stroke models, and the most common ones are cardiac arrest, the four-vessel occlusion model, and systemic hypotension and hypoxia. Animal models for focal ischemic strokes have been developed to induce damage within the territory irrigated by the middle cerebral artery (MCA) region to mimic a common clinical situation. There are different approaches to achieve this, but the most common ones are the intraluminal suture middle cerebral artery occlusion model without reperfusion (MCAO) and with reperfusion (MCAO/R), the photothrombotic model, and the endothelin-1 induced stroke model [112]. In this section, we will analyze only the studies that applied flavonoids after vessel occlusion with or without reperfusion because these models are more similar to clinical strokes. In a rat MCAO/R model, kaempferol-3-*O*-rutinoside (KRS) and kaempferol-3-*O*-glucoside (KGS) reduced the neurological deficits and infarct volume and inhibited the proinflammatory mediators (STAT3 and NF-κB) and interleukin 1β [113]. In the same model, kaempferol administered for 7 days after stroke also decreased the NF-κB and pro-inflammatory cytokines such as IL-5, TNF-α, IL-1β, and IL-6, but the reduction of last three cytokines only occurred with high doses [114]. Fisetin applied 3 h after the onset of ischemia to the MCAO/R mice model significantly reduced the infarct size and decreased TNF-α production in the microglia and the infiltration of leukocytes and macrophages [115]. A morin treatment for 7 days after MCAO in rats reduced the neurological deficits and inhibited the proinflammatory cytokine mRNA expression of TNF-α and IL-6 [116], and in the MCAO/R rat model, it decreased the rates of pNF-κB, TNF-α, IL-1β, and TLR4 expression and improved the tight junctions of the BBB by significantly increasing occludin and claudin expression [117]. (−)-Epigallocatechin-3-gallate (EGCG) decreased the infarct volume, TNF-α, IL-1β, and IL-6, and also, inhibited NF-κB/p65 in a MCAO/R rat model when it was applied immediately after reperfusion [118]. Luteolin administered intraperitoneally after MCAO/R in rats suppressed hippocampus inflammation, reduced the infarct volume, and decreased the astrocyte and microglia activation [119]. Luteoloside decreased the infarct volume, TNF-α, and IL-1β when this molecule was intraperitoneally injected immediately and 12 h after MCAO surgery in rats [120]. MCOA/R rats treated with nobiletin after reperfusion resulted in improved neurological deficits and decreased brain swelling and infarct volume [121]. Eriodictyol applied after stroke improves neurological deficits in the MCAO mice model and also decreases infarct volume TNF-α and GFAP expression [122]. Tricin applied by oral administration 2 h, 4 h, and 6 h after MCA/R resulted in decreased serum levels of TNF-α, IL-6, and IL-1β [109]. Eupatilin administered to mice 5 h after MCAO/R showed a reduction of the activated microglia in the peri-ischemic tissue and inhibited the NF-κB pathway [123].

##### Synergistic Effect of Flavonoids with rtPA

The simultaneous treatment of EGCG and rt-PA 4 h after MCAO significantly reversed the neurobehavioral deficit, brain infarction, cerebral edema, and blood–brain barrier disruption [124].

##### Preventive Treatment with Flavonoids

Rats treated with naringenin once daily for 21 days, and then subjected to MCOA/R, resulted in a significant decrease in the infarct volume, the expression of NF-κB, TNF-α, IL-1β, and GFAP in the astrocytes, and Iba1 in the microglia, and improved neurologic deficits [125]. Moreover, rats treated with the same molecule for 4 days before MCAO also showed a decrease in NF-κB, the infarct volume, and improvements to the neurologic deficits [126]. Moreover, the same molecule applied 7 days before MCAO/R in rats decreased the expression of TNF-α and IL-6 in the brain tissue [126]. Rats treated with hesperidin for 15 days followed by MCAO showed an improvement of the neurological deficits, infarct volume, and decreased levels of IL-1β [127]. In a rat model of global stroke, a pinocembrin treatment administered daily for 7 days before a stroke decreased the infarct size and NF-κB, TNF-α, and IL-6 in the hippocampal tissue [128]. In an MCAO mice model, a fourteen-day-long genistein treatment before a stroke reduced the infarct volume, improved the neurological deficit, and inhibited NF-κB activation [129]. Sanggenon administered intragastrically in rats seven days before MCAO/R surgery resulted in a decrease in the levels of TNF-α, IL-1β, and IL-6 [130]. Astilbin applied 3 days before the MCAO in rats inhibited NLRP3 inflammasome and decreased the serum concentration of IL-1β and IL-18 [107]. Chrysin applied for 7 days before the MCAO in rats decreased the release of inflammatory cytokines IL-6, IL-1β, and TNF-α [131]. Baicalin applied 4 days before the MCAO in rats decreased the expression level of the NLRP3 inflammasome, IL-1β, and IL-18 [97]. Eupafolin applied for 7 days before the MCAO/R in rats decreased TLR-4, TNF-α, IL-1β, and IL-6 expression [132]. Rats were treated with Biochanin A for 14 days before the MCAO in rats decreased the protein and gene expression of TNF-α and IL-1β [133].

#### 6.1.3. Clinical

Though no clinical studies using flavonoids as a post-stroke treatment could be found, some human studies suggest that flavonoids can be useful in the treatment of this condition. A study using the flavonol fisetin combined with rt-PA in stroke patients shows that the addition of this flavonoid extends the therapeutic window of rt-PA treatment and dramatically improves the neurological deficits evaluated by the National Institutes of Health Stroke Scale (NIHSS) and decreases the plasma levels of C-reactive protein (CRP) and matrix metalloproteinases (MMP)-2 and -9 [134]. In a similar study, EGCG also extends the therapeutic window of the rt-PA treatment and improves the NIHSS scale, while decreasing plasma levels of matrix metalloproteinases (MMP)-2 and -9 [135].

### 6.2. Anti-Inflammatory Effects of Flavonoids in AD

#### 6.2.1. In Vitro Studies

Numerous in vitro studies have evaluated the effects of different flavonoids in the Aβ oligomer and its assembly into aggregates. EGCG inhibits the fibrillogenesis of Aβ [136], modifies Aβ fibrils into smaller protein aggregates that are nontoxic to mammalian cells [137], and in cultured hippocampal neuronal cells, it has protective effects against Aβ-induced neuronal apoptosis through scavenging reactive oxygen species [138]. Quercetin used as a pretreatment with primary hippocampal cultures significantly decreases Aβ (1–42)-induced cytotoxicity, lipid peroxidation, protein oxidation, and apoptosis [139]. Luteolin and diosmetin decrease Aβ (1–40 and 1–42) in primary neuronal cells and SweAPP N2a cells [140]. Myricetin prevents the fibrillogenesis of Aβ [141]. Cyanidin 3-*O*-β-glucopyranoside in neuroblastoma SH-SY5Y cells reduces the cytotoxicity of Aβ (25–35) and its aggregation [142]. In the same cells, wogonin reduces Aβ aggregation and phosphorylated Tau [143]. Through different biochemical techniques, it has been found that Baicalein prevents the aggregation of the human tau protein [144,145]. Quercetin and rutin prevent the formation of Aβ fibrils and disaggregate Aβ fibrils in a cell system overexpressing APP Swedish mutation (APPswe) [146].

#### 6.2.2. Animal Model Studies

Animal models for AD include chemically induced ones (e.g., amyloid infusion and streptozotocin), spontaneous ones (e.g., senescence-accelerated mouse), and several transgenic mice and a few transgenic rats that express mutant human genes related to the production of amyloid plaques and neurofibrillary tangles (e.g., 3XTg and 5XFAD, TG2576, and APP/PS1) [147]. These transgenic animals model familial AD and partly recapitulate the idiopathic forms and they express amyloid plaques and neurofibrillary tangles and all of the manifested deficits in memory, but the majority the animals do not present with neurodegeneration, and this is one of the aspects that limits their use in neuroinflammation research [147,148]. Since these animal models do not present with neurodegeneration, the main interest of flavonoids in the research in these animal models has been focused on the effects of the Aβ aggregation and the impairment of the cognition functions. In the chemical mouse model of memory deficits induced by scopolamine, isorhamnetin inhibits learning and memory deficits, and also, induces an increase in brain-derived neurotrophic factor (BDNF) levels in the prefrontal cortex and hippocampus [149], while naringin and rutin improve memory [150]. In another chemical model (streptozotocin), kaempferol increases the density of intact neurons in the CA1 area of the hippocampus and improves memory [151]. Luteolin improves memory [152] and a hesperidin pretreatment decreases inflammatory markers, such as NF-κB, iNOS, COX-2, and astrogliosis, and improves memory [153]. Nobiletin in APP-SL 7-5 transgenic mice reduces the quantity of soluble Aβ (1–40 and 1–42) and Aβ plaques in the hippocampus [154]. In 3XTg mice, diosmin and its bioactive metabolites decrease tau hyperphosphorylation and Aβ generation [155]. In transgenic APP/PS1 mice, hesperidin reduces Aβ plaque in the cortex and the hippocampus, decreases astrogliosis and microglial activation, and restores the ability to perform social interaction [156]. In transgenic h-APPswe, h-Tau P301L, and h-PS1 M146V mice, wogonin improves memory [143]. In the Tg2576 transgenic mouse model, diosmin and luteolin reduce Aβ 1–40 and 1–42 [140]. In APPsw transgenic mice, EGCG reduces the amount of soluble Aβ (1–40 and 1–42) and Aβ deposits in different cortical brain regions and the hippocampus [157], and in the Aβ infusion model, it prevents memory dysfunction and reduces the Aβ (1–42) and alpha-secretase levels and increases beta- and gamma-secretase in both the cortex and hippocampus, and similar results were obtained in the presenilin 2 (PS2) mutant mice [158], while in the APPsw transgenic mouse model, diosmin shows improved memory [159]. Nobiletin reduces Aβ plaques in the hippocampus and improves memory deficits in APP-SL 7-5 transgenic mice [154], and in 3XTg mice, it reversed the damage to memory and decreased the levels of Aβ 1–40 [160]. Nobiletin reverses memory impairment in the hippocampus in senescence-accelerated mice SAMP8 [161]. In 3XTg mice, quercetin reduces the plaques of Aβ and hyperphosphorylated tau in the CA1 area of the hippocampus and improves memory [162]. Cyanidin 3-*O*-glucoside decreases tau phosphorylation in the hippocampus and reverses memory impairment in Aβ infusion rats [163] and in the APP(swe)/PS1(ΔE9) mouse model, it improved memory and learning [142]. Fisetin in APPswe/PS1dE9 double transgenic mice inhibits the development of memory and learning problems through the modulation of cyclin-dependent kinase 5 (Cdk5), where hyperactivity induces neuroinflammation and neurodegeneration [164]. In two different transgenic mouse models (TG2576 and TG-SwDI), a dihydromyricetin treatment improves exploratory and locomotor activities, decreases anxiety, improves memory, and reverses Aβ accumulation [165]. As you may note, the majority of the studies mentioned focused on the effects of flavonoids on amyloidopathy and cognitive deficits, while studies on tauopathy are scarce.

#### 6.2.3. Clinical

There are no clinical studies with a single flavonoid molecule, but we can illustrate the potential of these molecules using the results obtained with cocoa flavanol, which is a mixture of flavanols, mainly catechin and epicatechin. Cocoa flavanol consumption for 8 weeks improved cognitive functions in patients with mild cognitive impairment [166], and a double-blind study showed improved cognitive functions in aging subjects [167]. Moreover, the consumption of these flavanols by healthy 50–69-year-old subjects over 3 months improves the dentate gyrus functions evaluated by a high-resolution variant of functional magnetic resonance imaging (fMRI) and cognitive testing [168].

### 6.3. Anti-Inflammatory Effects of Flavonoids in PD

#### 6.3.1. In Vitro Studies

Some in vitro studies have evaluated the effects of different flavonoids on the formation of α-synuclein oligomers and their assembly into aggregates and found that flavonoids inhibit oligomer formation and aggregation. These flavonoids include: apigenin, baicalein myricetin, genistein, morin, quercetin, EGCG, and scutellarein [169,170,171]. In activated microglia induced by the LPS model, nobiletin prevents the release of the proinflammatory cytokines TNF-α and IL-1β [172], and apigenin and luteolin decrease TNF-α and IL-6 [173]. Naringenin inhibits NF-κB, iNOS, and COX-2, and induces the expression of the suppressor of cytokine signaling 3 (SOCS-2), a negative regulator of cytokines in activated microglia [174,175]. Diadzein downregulates the activation of NF-κB and the production of IL-6 and [176], and its metabolite Equol (7-hydroxy-3-(4′-hydroxyphenyl)-chroman) prevents the secretion of TNF-α, IL-6, and NF-κB activation [177]. In PC12 cells exposed to the neurotoxic MPP^+^, morin reduces cell apoptosis and mortality [178] and decreases the rate of astrogliosis and the nuclear translocation of NF-κB in primary cultured astrocytes exposed to the same neurotoxicity [179]. Butein, butin, fisetin, fustin, and sulfuretin protect the murine hippocampal HT22 cells against glutamate-induced neurotoxicity and reduce the induced nitric oxide (NO) production in BV2 cells microglial cell lines, and also, butein suppresses the expression of iNOS and COX-2 [180]. Genkwanin suppressed the MPP^+^-induced activation of the TLR4/MyD88/NLRP3 inflammasome pathway in SH-SY5Y cells [181].

#### 6.3.2. Animal Model Studies

Chemically induced animal models of PD are the most widely used ones. Rat or mice models of 1-methyl-4-phenyl-1,2,3,6-tetrahydropyridine (MPTP) and 6-hydroxydopamine (6-OHDA) are two of the most common types. MPTP is the precursor to the neurotoxic 1-methyl-4-phenyl-2,3-dihydropyridinium (MPDP^+^), which is converted into glial cells. Both MPDP^+^ and 6-OHDA are neurotoxic to dopaminergic neurons and elicit a motor phenotype [182,183]. Additionally, the rotenone model of PD also shows the main pathological hallmarks of the disease [184]. In this last model, baicalein reduced the formation and accumulation of α-synuclein oligomers and protected dopaminergic neurons [185]. In the MPP^+^ rat model, this flavonoid attenuates α-synuclein aggregation, inhibits inflammasome activation [186], improves the motor ability, decreases the number of activated microglia and astrocytes, and increases dopamine and serotonin neurotransmitters in the striatum [187,188,189]. Apigenin in the rotenone rat model decreases the expression of NF-κB, increases the expression of dopamine D2 receptor (D2R), and decreases α-synuclein aggregation in the rat rotenone model [190]. Nobiletin in the MPP^+^ rat model preserved the expression of the glial-cell-line-derived neurotrophic factor (GDNF), inhibited microglial activation [191] and increased the dopamine contents in the striatum and hippocampal CA1 region, and improved the motor deficits [192]. In the MPP^+^ rat model, naringin increased the GDNF level in the substantia nigra, reduced TNF-α expression [193], and protected the nigrostriatal DA projection [194]. In the MPDP^+^ mice model, EGCG reduced the dopamine neuronal loss in the substantia nigra [195], and the same flavonoid in the rotenone rat model inhibited TNF-α, IL-1β, and IL-6 in the striatum [196]. Quercetin increased the striatal dopamine level and reduced dopaminergic neuronal loss in the 6-OHDA rat model [197]. The same flavonoid in the MitoPark transgenic mouse models of PD reversed dopaminergic neuronal loss, striatal dopamine depletion, and the behavioral deficits [198]. Quercetin and kaempferol in an MTPT mouse model improved striatal dopamine secretion and motor coordination [199,200]. In the same model, hesperidin reduced the expression of IL-1β, TNF-α, and IL-6 and improved motor coordination [201,202]. In the 6-OHDA rat model, tangeretin protected the striatal dopaminergic neurons [203], rutin protected dopaminergic neurons and improved motor coordination [204], troxerutin reduced neuronal loss, astrogliosis, and striatal lipoperoxidation [205], and myricitrin inhibited the expression of TNF-α and protected the dopaminergic neurons from the substantia nigra [206]. In the MPTP mouse model, a pretreatment with morin reduced dopaminergic neuronal death, behavioral deficits, and striatal dopamine depletion [178]; it reduced the dopaminergic neuronal losses, astrogliosis, and improved motor dysfunction [179]. In the MPTP mouse model, icariin inhibited NLRP3 inflammasome and decreased the IL-1β and TNF-α serum levels [207]. Europinidin in the rotenone rat model decreased the IL-6, IL-1β, and TNF-α levels in the brain tissue [208]. Diosmin in a rotenone rat model decreased the expression of NF-κB and the TNF-α levels [209].

#### 6.3.3. Clinical

We did not find any clinical study using a single flavonoid molecule, but research using flavonoid-rich pure cocoa in patients with PD could illustrate the possible benefits for this disease. A randomized (1:1), double-blind, placebo-controlled feasibility study with 30 patients with PD showed a reduction of fatigue and fatigability [210].

Table 1, Table 2 and Table 3 show the main anti-inflammatory effects in models of focal ischemic stroke, AD, and PD.

## 7. Conclusions and Future Directions

**Figure 3:**
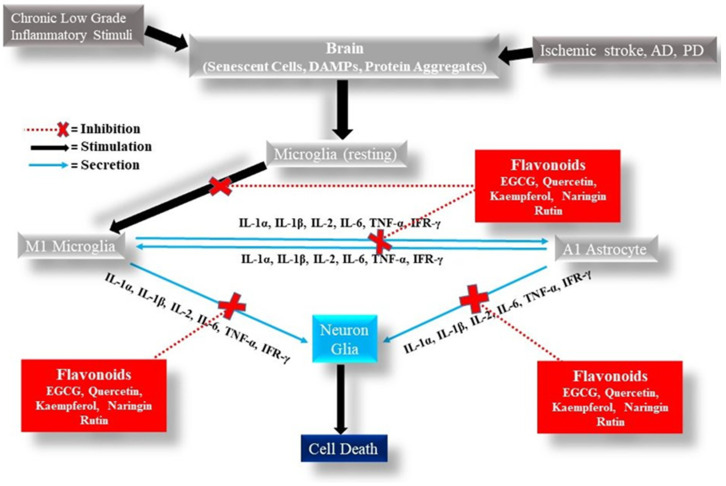
Anti-inflammatory effect of flavonoids. Flavonoids act as inhibitors of the activity of several transcription factors and regulatory enzymes to decrease the synthesis and release of different the pro-inflammatory cytokines.

Flavonoids have pleiotropic effects, which are demonstrated mainly through in vitro and animal models of diverse human diseases. The best-known mechanism is antioxidants, but in the last two decades, our knowledge of their effects on neuroinflammation has grown. Many in vitro and animal model studies highlight the anti-inflammatory effect of flavonoids by decreasing the activated microglia and astrocytes, and also, by decreasing the proinflammatory cytokines such as TNF-α, IL-1β, IL-6, COX-2, and iNOS, either by the indirect or direct inactivation of transcription factors such as NF-κB and AP-1, and also, through the inactivation of the inflammasome (Figure 3). On the other hand, several studies have found that flavonoids can downregulate other pathological pathways, and some human studies show that after consuming flavonoid-rich foods and beverages, there is a significant reduction of the proinflammatory molecules, including C-reactive protein and IL-6 [211,212,213]. Searching the clinical trial data set from ClinicalTrails.gov revealed the interest of the scientific community in the beneficial effects of flavonoids for human health, as several ongoing studies are focused on topics related to ischemic strokes and neurodegenerative diseases, such as cognition performance, cognitive aging, the risk of dementia, and endothelial dysfunction, suggesting a growing interest in translating the preclinical knowledge into clinical trials.

Although there have been substantial achievements in the bioavailability of flavonoids, for example, the cutaneous delivery system and application of the nanoencapsulation of bioactive compounds [214,215], we still need to improve our knowledge about aspects such as metabolic transformation, the identification of active molecules (parent molecule and/or their metabolites), the mechanism to cross the blood-brain barrier, and toxicology to find single flavonoids for the future therapy for neurological disorders associated with aging.

## Figures and Tables

**Table 1:** Anti-inflammatory effects of flavonoids in focal ischemic stroke models and clinic studies.

**Flavonoid**	**Effect**	**Model (In Vitro)**	**References**
Myricetin	↓ TNF-α, IL-1β, and IL-6	OGD/R, endothelial cells	[92]
Quercetin	↓ TLR4/MyD88/NF-κB signaling	BV2 microglial cells	[93]
Isoquercetin	↓ TLR4, NF-κB, TNF-α, and IL-6	OGD/R, neurons	[94,95]
Baicalin	↓ TNFα and NOD2 receptor	OGD, BV2 microglial cells	[96]
Baicalin	↓ NLRP3 inflammasome	OGD/R, cortical neurons	[97]
Baicalin	↓ TNF-α, IL-1β, and IL-6	OGD/R, endothelial cells	[98]
Baicalin	↓ TNF-α, IL-1β, and IL-6	OGD/R, BV2 microglia cell	[99]
Baicalin	↓ TLR4/NF-κB pathway	microglia-neuron co-culture	[100]
Baicalin	↓ TLR4, MYD88, p-NF-κB expression, ↓ IL-6, IL-1α, IL-1β, IL8, and TNF-α	OGD, endothelial cells	[101,102]
Icariin	↓ IL-1β, IL-6, and TNF-α expression	OGD/R, microglia	[103]
Casticin	↓ TLR4, NF-κB p65, NF-κB, and p50 expression	OGD/R, PC12 cells	[104]
Pratensein	↓ NLRP3 inflammasome	OGD/R, HT22 cells	[105]
Tectorigenin	↓ IL-1β, IL-6, and TNF-α	OGD/R, HT22 cells	[106]
Astilbin	↓ NLRP3 inflammasome, IL-1β, and IL-18	OGD, PC12 cells	[107]
Anthocyanin	↓ TNF-α, IL-1β, and IL-6	OGD, SH-SY5Y cells	[108]
Tricin	↓ TNF-α and IL-6 and ↓ IL-1β expression	Neuroblastoma cells	[109]
Diosmetin	↓ NLRP3 inflammasome	OGD/R, PC12 cells	[110]
Schaftoside	↓ TLR4, IL-1β, IL-6, and ↓ TNFα expression	OGD, BV2 microglia	[111]
**Flavonoid**	**Effect**	**Model (In Vivo)**	**References**
Kaempferol-3-*O*-rutinoside and 3-*O*-glucoside	↓ infarct volume, STAT3, NF-κB and IL-1β	MCAO/R, rat	[113]
Kaempferol	↓ IL-5, TNF-α, IL-1β, and IL-6	MCAO/R, rat	[114]
Fisetin	↓ infarct size, TNFa	MCAO/R, mouse	[115]
Morin	↓ neurological deficits, TNF-α, and IL-6	MCAO/R, rat	[116]
Morin	↓ pNF-κB, TNF-α, and IL-1β, TLR4 expression, ↑ occluding, claudin expression	MCAO/R, rat	[117]
EGCG	↓ infarct volume, TNF-α, IL-1β, IL-6, and NF-κB/p65	MCAO/R, rat	[118]
Luteolin	↓ infarct volume, astrocytes and microglia activation	MCAO/R, rat	[119]
Luteoloside	↓ infarct volume, TNF-α, and IL-1β	MCAO, rat	[120]
Nobiletin	↓ infarct volume, brain swelling, and neurological deficits	MCOA/R, rat	[121]
Eriodictyol	↓ infarct volume, neurological deficits, TNF-α, and GFAP expression	MCAO, mouse	[122]
Tricin	↓ TNF-α, IL-6, and IL-1β in serum	MCAO, mouse	[[109]
Eupatilin	↓ microglia activation and NF-κB pathway	MCAO, mouse	[123]
EGCG + rt-PA	↓ neurobehavioral deficit, brain infarction, cerebral edema, and blood-brain barrier disruption	MCAO, rat	[124]
**Flavonoid**	**Effect (Preventive Treatment)**	**Model (In Vivo)**	**References**
Naringenin	↓ infarct volume, NF-κB, TNF-α, IL-1β, GFAP, and Iba1	MCOA/R, rat	[125]
Naringenin	↓ infarct volume, neurologic deficits, and NF-κB	MCOA/R, rat	[126]
Hesperidin	↓ IL-1β	MCOA, rat	[127]
Pinocembrin	↓ infarct size and NF-κB, TNF-α, and IL-6	Global stroke, rat	[128]
Genistein	↓ infarct volume, neurological deficit, and NF-κB activation	MCAO, mouse	[129]
Sanggenon	↓ TNF-α, IL-1β, and IL-6	MCOA, rat	[130]
Astilbin	↓ NLRP3 inflammasome, IL-1β, and IL-18	MCOA, rat	[107]
Chrysin	↓ IL-6, IL-1β, and TNF-α	MCOA, rat	[131]
Eupafolin	↓ TLR-4, TNF-α, IL-1β, and IL-6 expression	MCOA/R, rat	[132]
Biochanin A	↓ TNF-α and IL-1β expression	MCOA, rat	[133]
**Flavonoid**	**Effect**	**Clinical Studies**	**References**
fisetin + rt-PA	↑ therapeutic window of rt-PA and ↓ neurological deficits, C-reactive protein	Double-blind randomized placebo-controlled	[134]
EGCG + rt-PA	↑ therapeutic window of rt-PA, ↓ MMP 2, and MMP 9	Double-blind randomized placebo-controlled	[135]

↑: significant increase; ↓: significant decrease; TNF-α: tumor necrosis factor α; IL: interleukin; TLR4: Toll-like receptor 4; NF-κB: nuclear factor kappa B; rtPA: recombinant tissue plasminogen activator; OGD: oxygen glucose deprivation; OGD/R: oxygen glucose deprivation with reperfusion; MCAO: middle cerebral artery occlusion; MCAO/R: middle cerebral artery occlusion with reperfusion; MMP: matrix metalloproteinase.

**Table 2:** Anti-inflammatory effects of flavonoids in AD models and clinical studies.

**Flavonoid**	**Effect**	**Model (In Vitro)**	**References**
EGCG	Prevents Aβ fibrillogenesis and ↓ cell toxicity	Protein aggregation, PC12 cells	[136,137]
EGCG	Protective against Aβ toxicity	Hippocampal neuronal cell culture	[138]
Quercetin	↓ Aβ cytotoxicity, lipid peroxidation, protein oxidation, and apoptosis	Hippocampal neuronal cell culture	[139]
Luteolin	↓ Aβ (1–40 and 1–42)	Neuronal cells and SweAPP N2a cells	[140]
Diosmetin	↓ Aβ (1–40 and 1-42)	Neuronal cells and SweAPP N2a cells	[140]
Myricetin	Prevents Aβ fibrillogenesis	Cerebral cortices from Tg2576 mouse embryos	[141]
Cyanidin 3-*O*-β-glucopyranoside	↓ Aβ (25–35) cytotoxicity	SH-SY5Y cells	[142]
Wogonin	↓ Aβ aggregation and phosphorylated Tau	SH-SY5Y cells	[143]
Baicalein	Prevents tau protein aggregation	Several biochemical techniques	[144,145]
Quercetin	Prevent Aβ aggregation and ↑ disaggregate	Cell system overexpressing APP	[146]
Rutin	Prevent Aβ aggregation and ↑ disaggregate	Cell system overexpressing APP	[146]
**Flavonoid**	**Effect**	**Model (In Vivo)**	**References**
Isorhamnetin	↓ learning and memory deficits and ↑ BDNF in prefrontal cortex and hippocampus	Chemical mouse model	[149]
Naringin	Improve memory	Chemical rat model	[150]
Rutin	Improve memory	Chemical rat model	[150]
kaempferol	Improve memory and ↑ density of neurons in hippocampus	Chemical rat model	[151]
Luteolin	Improve memory	Chemical rat model	[152]
Hesperidin	improves memory and ↓ NF κB, iNOS, COX-2, and astrogliosis	Chemical mouse model	[153]
Nobiletin	↓ soluble Aβ (1–40 and 1–42) and Aβ plaques in the hippocampus	APP-SL 7-5 transgenic mouse	[154]
Diosmin and its bioactive metabolites	↓ tau hyperphosphorylation and Aβ generation	3xTg transgenic mouse	[155]
Hesperidin	↓ Aβ plaque in cortex and hippocampus and ↓ astrocyte and microglial activation	Transgenic APP/PS1 mouse	[156]
Wogonin	Improve memory	Transgenic h-APPswe mouse	[143]
Diosmin	↓ Aβ 1–40 and 1–42	Tg2576 transgenic mouse	[140]
Luteolin	↓ Aβ 1–40 and 1–42	Tg2576 transgenic mouse	[140]
EGCG	↓ soluble Aβ (1–40 and 1–42) and Aβ plaques in cortex and hippocampus	APPsw transgenic mouse	[157]
EGCG	↓ Aβ (1–42)	Aβ infusion model, presenilin 2 mutant mouse	[158]
Diosmin	Improve memory	APPsw transgenic mouse	[159]
Nobiletin	↓ Aβ plaques in the hippocampus and ↓ memory deficits	APP-SL 7-5 transgenic mouse	[154]
Nobiletin	↓ memory impairment, ↓ the levels of Aβ 1–40	3XTg transgenic mouse	[160]
Nobiletin	↓ memory impairment	Senescence-accelerated mice SAMP8	[161]
Quercetin	↑ memory and ↓ plaques of Aβ and hyperphosphorylated tau in hippocampus	3XTg transgenic mouse	[162]
Cyanidin 3-*O*-glucoside	↓ memory impairment, ↓ hyperphosphorylated tau in hippocampus	Aβ infusion rats	[163]
Fisetin	↓ memory and learning problems	APP(swe)/PS1(ΔE9) mouse	[164]
Dihydromyricetin	↑ exploratory and locomotor activity, and memory, ↓ anxiety and Aβ accumulation	TG2576 and TG-SwDI mouse	[165]
**Flavonoid**	**Effect**	**Clinical Studies**	**References**
Cocoa flavanol	Improves cognitive function	Patients with mild cognitive impairment	[166]
Cocoa flavanol	Improved cognitive function in aging subjects	Double-blind study	[167]
Cocoa flavanol	Improves dentate gyrus functions	fMRI in healthy 50–69-year-old subjects	[168]

↑: significant increase; ↓: significant decrease; TNF-α: tumor necrosis factor α; IL: interleukin; TLR4: Toll-like receptor 4; NF-κB: nuclear factor kappa B; Aβ: amyloid-beta; iNOS: inducible nitric oxide synthase; COX-2: cyclooxygenase 2; fMRI: functional magnetic resonance imaging.

**Table 3:** Anti-inflammatory effects of flavonoids in PD models and clinical studies.

**Flavonoid**	**Effect**	**Model (In Vitro)**	**References**
Apigenin	Inhibit oligomer formation and aggregation of α-synuclein	Protein aggregation	[169]
Baicalein	Inhibit oligomer formation and aggregation of α-synuclein	Protein aggregation	[169]
Myricetin	Inhibit oligomer formation and aggregation of α-synuclein	Protein aggregation	[170]
Morin	Inhibit oligomer formation and aggregation of α-synuclein	Protein aggregation	[169]
Genistein	Inhibit oligomer formation and aggregation of α-synuclein	Protein aggregation	[169]
Quercetin	Inhibit oligomer formation and aggregation of α-synuclein	Protein aggregation	[169]
EGCG	Inhibit oligomer formation and aggregation of α-synuclein	Protein aggregation	[171]
Scutellarein	Inhibit oligomer formation and aggregation of α-synuclein	Protein aggregation	[169]
Nobiletin	↓ TNF-α and IL-1β	Activated microglia	[172]
Apigenin	↓ TNF-α and IL-6	Activated microglia	[173]
Luteolin	↓ TNF-α and IL-6	Activated microglia	[173]
Naringenin	↓ NF-κB, iNOS, and COX-2	Activated microglia	[174,175]
Diadzein	↓ NF-κB and IL-6	Activated microglia	[176]
Equol	↓ TNF-α, IL-6, and NF-κB	Activated microglia	[177]
Morin	↓ cell apoptosis and mortality	PC12 cells exposed to MPP^+^	[178]
Morin	↓ astrogliosis and NF-κB	Astrocytes exposed to MPP^+^	[179]
Butein	↓ toxicity	HT22 and Microglial BV2 cells exposed to glutamate	[180]
Butin	↓ toxicity	HT22 and Microglial BV2 cells exposed to glutamate	[180]
Fisetin	↓ toxicity	HT22 and Microglial BV2 cells exposed to glutamate	[180]
Fustin	↓toxicity	HT22 and Microglial BV2 cells exposed to glutamate	[180]
Sulfuretin	↓ toxicity	HT22 and Microglial BV2 cells exposed to glutamate	[180]
Genkwanin	↓ TLR4/MyD88/NLRP3 inflammasome pathway	SH-SY5Y cells	[181]
**Flavonoid**	**Effect**	**Model (In Vivo)**	**References**
Baicalein	↓ α-synuclein	Rotenone	[185]
Baicalein	↓ α-synuclein and inflammasome	MPP^+^ rat	[186]
Baicalein	↑ motor ability, ↓ activated microglia and astrocytes, and ↑ dopamine and serotonin in the striatum	MPP^+^ rat	[187,188,189]
Apigenin	↓ α-synuclein and NF-κB	Rotenone rat	[190]
Nobiletin	↓ microglial activation	MPP^+^ rat	[191]
Nobiletin	↑ dopamine in the striatum and hippocampal region	MPP^+^ rat	[192]
Naringin	↑ GDNF in the substantia nigra	MPP^+^ rat	[193]
Naringin	Protects the nigrostriatal DA projection	MPP^+^ rat	[194]
EGCG	↓ dopamine neuronal loss	MPP^+^ mouse	[195]
EGCG	↓ TNF-α, IL-1β, and IL-6 in the striatum	MPP^+^ rat	[196]
Quercetin	↑ dopamine in the striatum, ↓ dopamine neuronal loss	6-OHDA rat	[197]
Quercetin	↓ dopaminergic neuronal loss and behavioral deficits	MitoPark transgenic mouse	[198]
Quercetin	↑dopamine and motor coordination	MTPT mouse	[199,200]
Kaempferol	↑dopamine and motor coordination	MTPT mouse	[199,200]
Hesperidin	↑ motor coordination and ↓ TNF-α, IL-1β, and IL-6	MTPT mouse	[201,202]
Tangeretin	Protects the striatal dopaminergic neurons	6-OHDA rat	[203]
Rutin	↑ motor coordination and dopaminergic neurons	6-OHDA rat	[204]
Troxerutin	↓ neuronal loss and astrogliosis	6-Hydroxydopamine lesion, rat	[205]
Myricitrin	Protects the striatal dopaminergic neurons and ↓ TNF-α	6-Hydroxydopamine lesion, rat	[206]
Morin	↓ neuronal loss and behavioral deficits	MTPT mouse	[178]
Morin	↑ motor coordination and ↓ dopamine neuronal loss	MTPT mouse	[179]
Icariin	↓ NLRP3 inflammasome, IL-1β, and TNF-α in serum	MTPT mouse	[207]
Europinidin	↓ IL-6, IL-1β, and TNF-α	Rotenone rat	[208]
Diosmin	↓ TNF-α and NF-κB	Rotenone rat	[209]
**Flavonoid**	**Effect**	**Clinical Studies**	**References**
Cocoa flavanol	↓ fatigue and fatigability	Double-blind placebo-controlled	[210]

↑: significant increase; ↓: significant decrease; TNF-α: tumor necrosis factor α; IL: interleukin; TLR4: Toll-like receptor 4; NF-κB: nuclear factor kappa B; iNOS: inducible nitric oxide synthase; COX-2: cyclooxygenase 2; MPTP: 1-methyl-4-phenyl-1,2,3,6-tetrahydropyridine; 6-OHDA: 6-hydroxydopamine: MPDP^+^: 1-methyl-4-phenyl-2,3-dihydropyridinium.

## Data Availability

Not applicable.
